# Job vacancies at the European Centre for Disease Prevention and Control (ECDC)

**DOI:** 10.2807/1560-7917.ES.2024.29.43.2410245

**Published:** 2024-10-24

**Authors:** Eurosurveillance editorial team

**Affiliations:** 1 European Centre for Disease Prevention and Control (ECDC), Stockholm, Sweden

The European Centre for Disease Prevention and Control (ECDC) invites applications for the following positions: 

**Scientific Officer Respiratory Viruses and Legionella**
The application deadline expires on 22 Nov 2024.

 **Principal Expert Respiratory Viruses**

The application deadline expires on 28 Nov 2024.
For more information, please visit the ECDC website:
https://www.ecdc.europa.eu/en/about-us/work-with-us/recruitment

